# Supplementary material for: Vinylene-Linked Emissive Covalent Organic Frameworks for White-Light-Emitting Diodes
Source: Polymers (Basel). 2023 Sep 8;15(18):3704. doi: 10.3390/polym15183704 (PMC10535042; doi:10.3390/polym15183704)
Supplement: Supplementary file 1 [file polymers-15-03704-s001.zip › polymers-2568117-supplementary.pdf]

polymers

MDPI

Supplementary Material

Vinylene-Linked Emissive Covalent Organic Frameworks for White-Light-Emitting Diodes

Yan Li, Xiaohan Wu, Jinyi Zhang, Congcong Han, Mengmeng Cao, Xiangrong Li and Jieqiong Wan

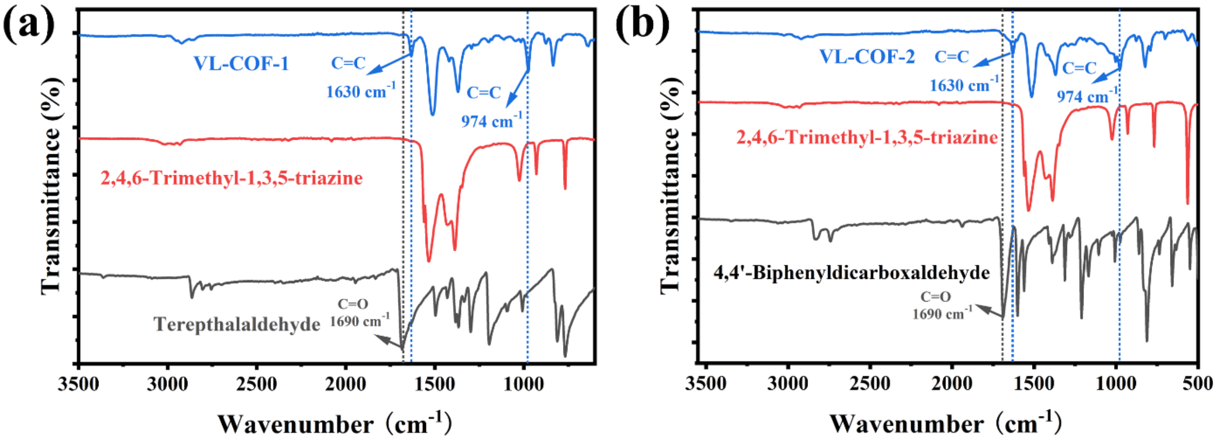

Figure S1. FTIR spectra of (a) VL-COF-1 (blue), 2,4,6-trimethyl-1,3,5-triazine (red), and terepthalaldehyde (black); FT IR spectra of (b) VL-COF-2 (blue), 2,4,6-trimethyl-1,3,5-triazine (red), and 4,4'-biphenyldicarboxaldehyde (black).

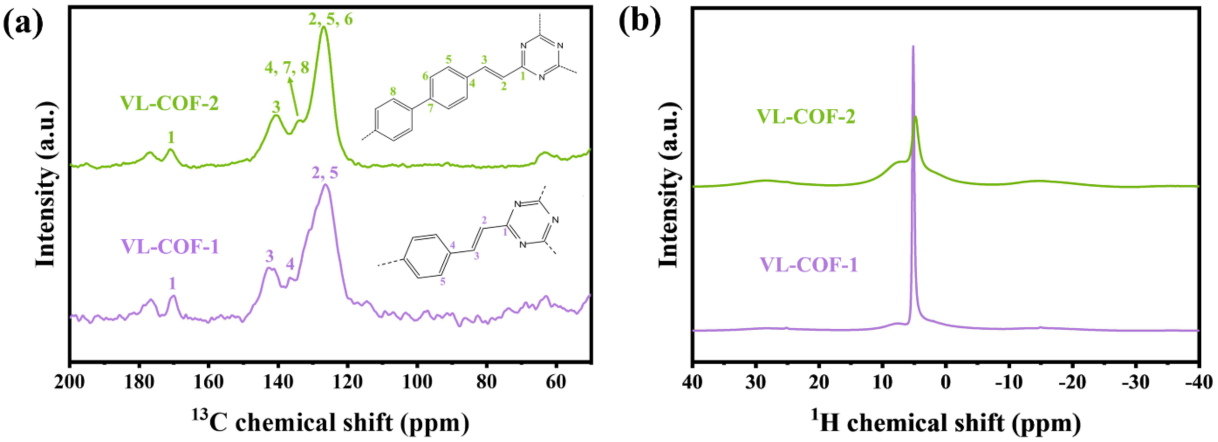

Figure S2. (a) ^{13}C CP/MAS solid-state NMR spectra of VL-COF-1 and VL-COF-2; (b) ^{1}H MAS solid-state NMR spectra of VL-COF-1 and VL-COF-2.

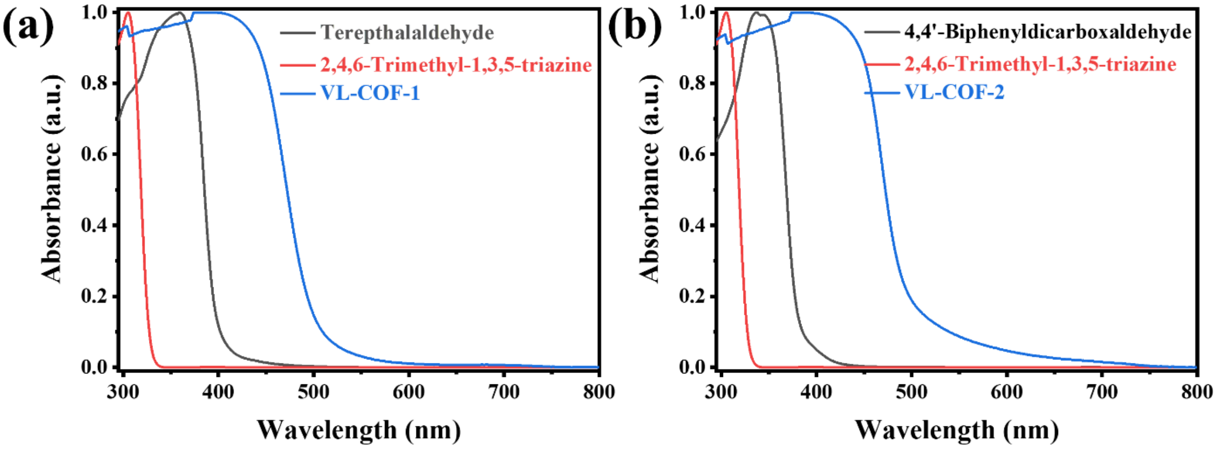

Figure S3. Normalized absorption spectra of (a) VL-COF-1 (blue), 2,4,6-trimethyl-1,3,5-triazine (red), and terepthalaldehyde (black); Normalized absorption spectra of (b) VL-COF-2 (blue), 2,4,6-trimethyl-1,3,5-triazine (red), and 4,4'-biphenyldicarboxaldehyde (black).

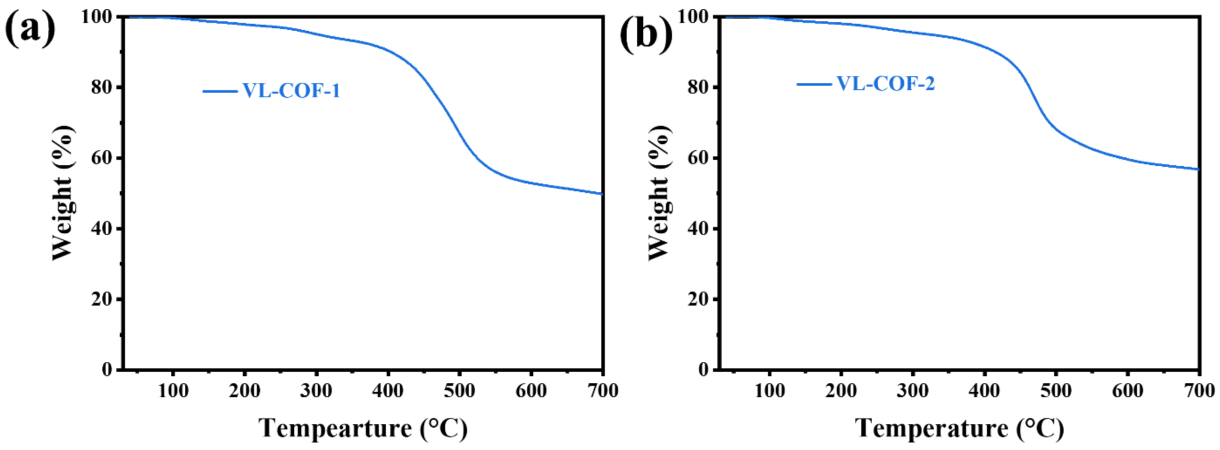

Figure S4. TGA curves of (a) VL-COF-1 and (b) VL-COF-2.

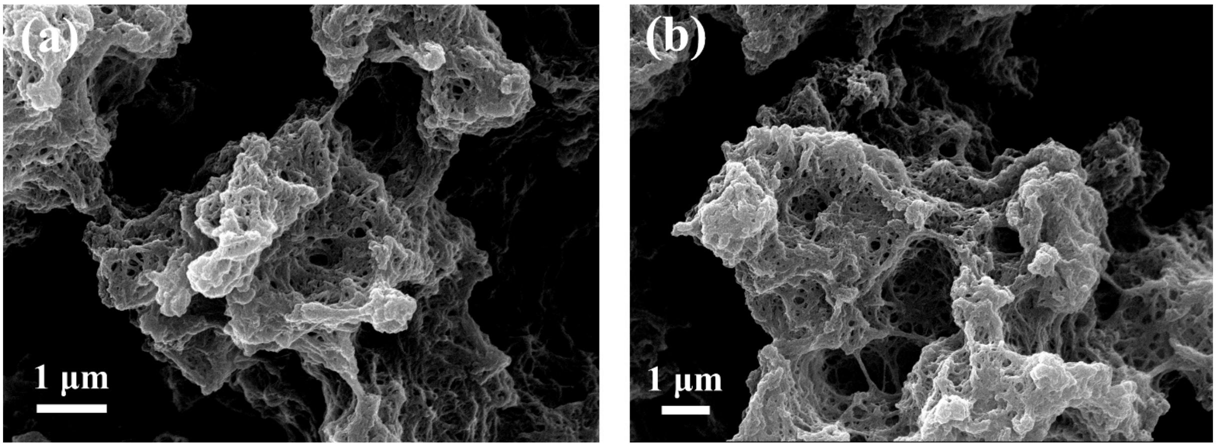

Figure S5. FE SEM images of (a) VL-COF-1 and (b) VL-COF-2.

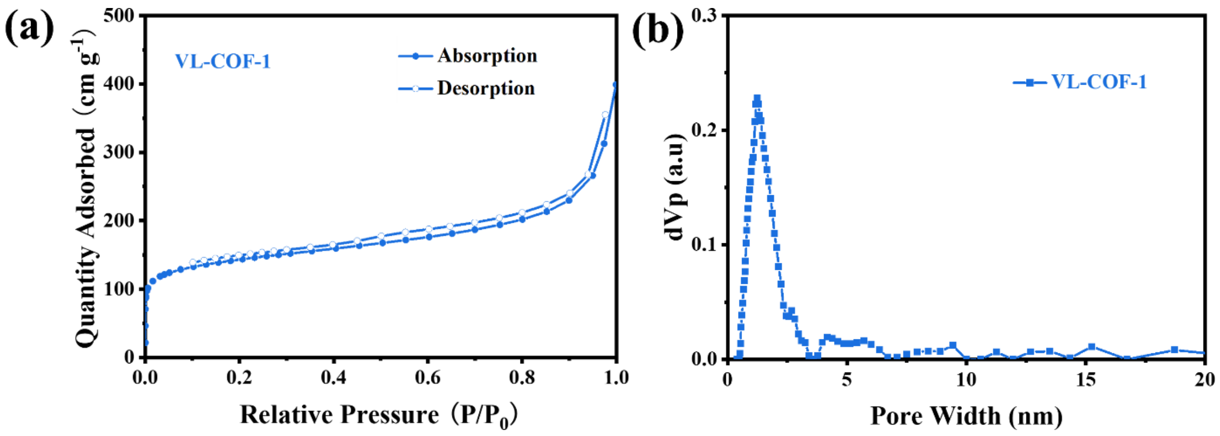

Figure S6. (a) Nitrogen adsorption-desorption isotherms of VL-COF-1 measured at 77 K; (b) Pore size distribution profile of VL-COF-1.

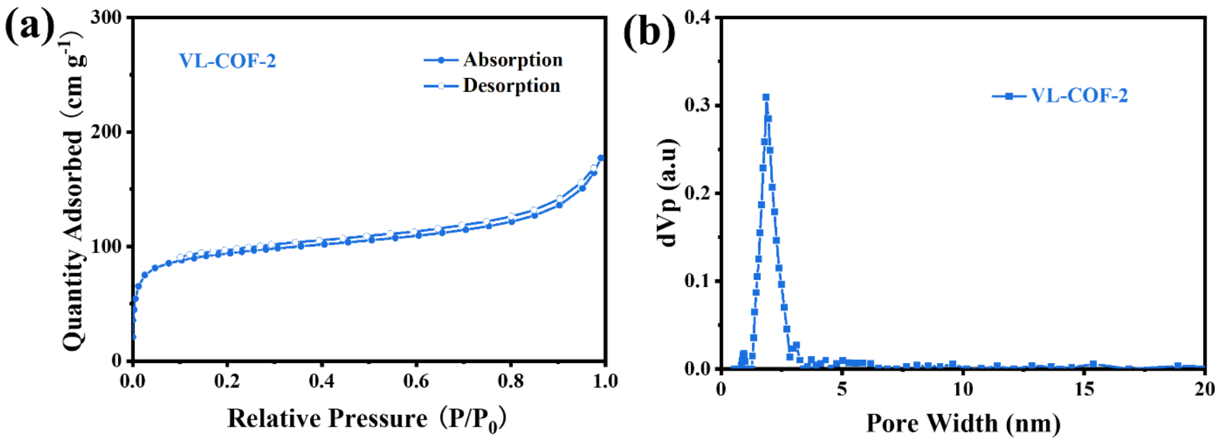

Figure S7. (a) Nitrogen adsorption-desorption isotherms of VL-COF-2 measured at 77 K; (b) Pore size distribution profile of VL-COF-2.

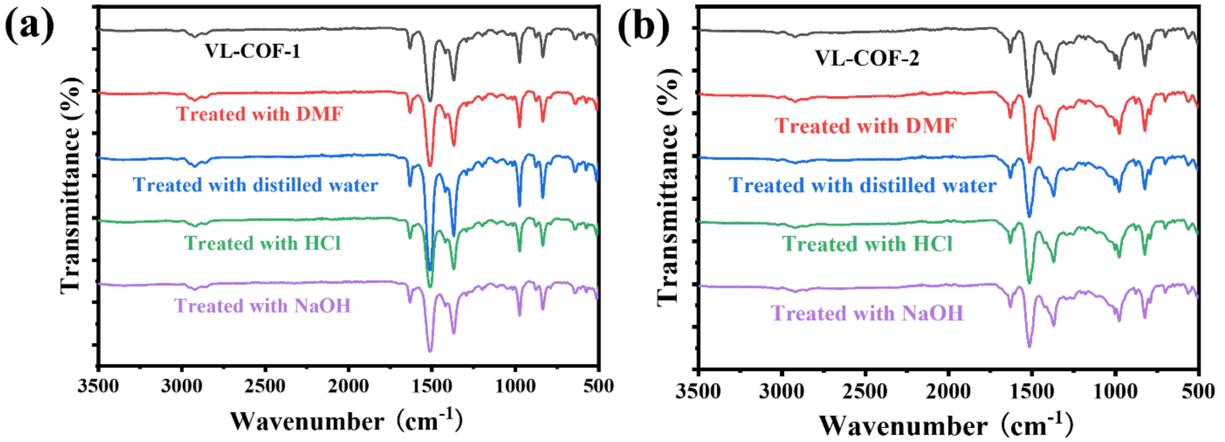

Figure S8. FTIR spectra of (a) VL-COF-1 and (b) VL-COF-2. (As synthetized VL-COFs: black; After post-treatment in DMF: red; Distilled water: blue; 6 M HCl solution: green; 6 M NaOH solution: purple.

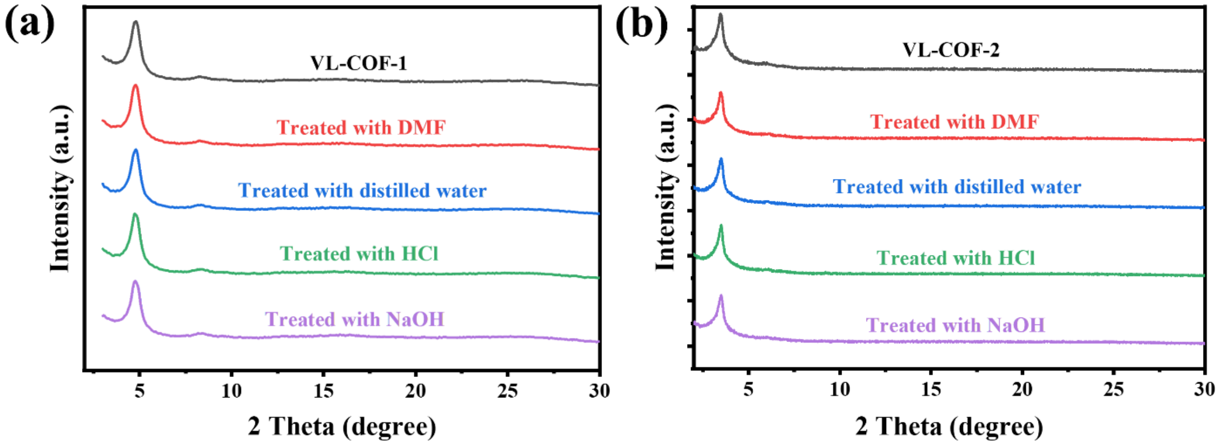

Figure S9. PXRD patterns of (a) VL-COF-1 and (b) VL-COF-2. (As synthetized VL-COFs: black; After post-treatment in DMF: red; Distilled water: blue; 6 M HCl: green; 6 M NaOH: purple.

Table S1. Representative summary of luminescence property of our COFs and literature–reported covalent organic frameworks in solid state.

COFs	Excitation Peak (nm)	Emission Peak (nm)	Fluorescence Lifetime (ns)	Quantum Yield (%)	Ref.
VL-COF-1	430	550	10.12	12.7	This work

VL-COF-2	445	525	7.51	11.2	
3D-TPE-COF	450	543	--	20	[12]
sp^2c-COF	497	622	1.85	14	
sp^2c-COF-2	475	606	2.93	10	[41]
sp^2c-COF-3	462	609	3.43	6	
TFPB-DHzDS	386	503	4.96	16.3	
TFPB-DHzDPr	378	474	2.69	14.4	[49]
Tf-DHzDPr	388	456	1.35	11.9	
Tf-DHzDM	390	456	1.78	8.2	
COF-LZU8	390	460	--	3.5	[50]
IMDEA-COF–1	365	501	44.70	3.5	[51]
PI–CON	375	500	5.28	8	[52]
TFPPy-DETHz-COF	460	540	1.4	4.5	[53]
COF-JLU3	424	601	1.5	9.91	[33]
